# Epstein-Barr Virus Masking Acalculous Cholecystitis in a 19-Year-Old Male

**DOI:** 10.7759/cureus.50508

**Published:** 2023-12-14

**Authors:** Fadi Barkho, Ikken Aisin, Andrew Korman

**Affiliations:** 1 Internal Medicine, Rutgers Robert Wood Johnson Medical School, New Brunswick, USA; 2 Internal Medicine, Saint Peter's University Hospital, New Brunswick, USA; 3 Gastroenterology and Hepatology, Saint Peter's University Hospital, New Brunswick, USA

**Keywords:** hepatobiliary, infectious mononucleosis, acalculous cholecystitis, ebv hepatitis, epstein-barr virus

## Abstract

Epstein-Barr virus (EBV), a member of the Herpesviridae family, is widely distributed and highly prevalent worldwide. It is known to cause infectious mononucleosis, characterized by symptoms such as fever, pharyngitis, lymphadenopathy, and atypical lymphocytosis in adults. In the pediatric population, acute EBV infection is typically asymptomatic. Acute cholecystitis, on the other hand, refers to acute inflammation within the gallbladder, typically due to the obstruction of the cystic duct secondary to gallstones. Patients will often present with right upper quadrant pain positive for Murphy sign, among other manifestations such as fever, fatigue, and jaundice. EBV and acute cholecystitis rarely coincide with one another. This abstract presents a comprehensive analysis of a clinical case that illustrates how an EBV infection obscured the clinical presentation of acute cholecystitis. This case underscores the necessity for a nuanced approach to the diagnosis, especially when the standard of care diagnostic criteria become inconclusive. However, hepatocellular injury as a result of EBV infection is rare. In this case report, we present the case of a 19-year-old male who developed EBV-induced hepatitis, emphasizing the importance of considering EBV as a potential cause in similar clinical scenarios.

## Introduction

Epstein-Barr Virus (EBV), a ubiquitous member of the herpesvirus family, is known for its diverse clinical presentations, often ranging from innocuous infectious mononucleosis to severe complications such as lymphoproliferative disorders and viral hepatitis. However, EBV's role in disguising and complicating the clinical manifestation of various underlying diseases remains an intriguing and evolving aspect of medical research. This introduction focuses on a fascinating and relatively underexplored facet of EBV's clinical impact - its potential to mask acute cholecystitis, an inflammatory condition of the gallbladder. Acute cholecystitis, typically characterized by right upper quadrant pain, fever, and localized tenderness, is a common disorder in clinical practice. Its diagnosis traditionally relies on a combination of clinical symptoms, laboratory investigations, and imaging studies. However, EBV infection can complicate this diagnosis, causing elevated alkaline phosphatase levels that mimic acute cholecystitis. This can lead to delayed EBV recognition, unnecessary surgical consultations, and costly diagnostic tests, ultimately burdening hospital systems. The enigmatic interplay between EBV and acute cholecystitis raises crucial questions: How does EBV alter the clinical presentation of cholecystitis? What are the underlying mechanisms behind this phenomenon? How can healthcare providers navigate this diagnostic challenge effectively? This inquiry into EBV's role in masking acute cholecystitis seeks to shed light on these intricate matters, emphasizing the significance of early and accurate diagnosis in optimizing patient care.

## Case presentation

A previously healthy 19-year-old male presented to the ED with a four-day history of epigastric abdominal pain, nausea, vomiting, fever, chills, malaise, fatigue, and decreased appetite. His symptoms worsened with food intake and deep breathing. He did not have bloody or green vomitus, constipation, recent travel history, or previous illness. Upon presentation, his vital signs were stable, and he was afebrile. His white blood cell count was normal, but he exhibited thrombocytopenia. He disclosed being sexually active with a single partner for over a year and had tested negative for sexually transmitted diseases one month ago. He denied smoking, consuming alcohol, using marijuana, or engaging in IV drug use. There were no relevant findings in his family history. Physical examination revealed non-tender submandibular lymphadenopathy bilateral without clustering or skin changes, hepatosplenomegaly, and tenderness in the right upper quadrant. Laboratory investigations on day 1 indicated elevated liver enzymes, with his aspartate aminotransferase at 150 U/L, alanine aminotransferase at 218 U/L, and total bilirubin at 1.6 mg/dL. Over the next five days, the values were noted to increase daily, as seen in Table 1. Hepatitis panel screening yielded negative results. The Monospot test was positive. Serological tests confirmed the presence of IgM antibodies for EBV, indicating an acute EBV infection. 

**Table 1:** Laboratory values during the course of hospital stay.

	Day 1	Day 2	Day 3	Day 4	Day 5	Day 6
Total bilirubin (n=0.1-1.2 mg/dL)	1.6	1.1	2.6	2.8	2.7	3.2
Alkaline phosphatase (n=53-128 U/L)	121	132	141	142	157	198
Aspartate aminotransferase (n=17-59 U/L)	159	167	317	718	770	586
Alanine transaminase (n=0-50 U/L)	218	232	362	652	882	792
Monospot test					Positive	
Epstein-Barr Virus IgM					88.1 copies/mL	
Epstein-Barr Virus DNA PCR					400,668 copies/mL	

**Figure 1:**
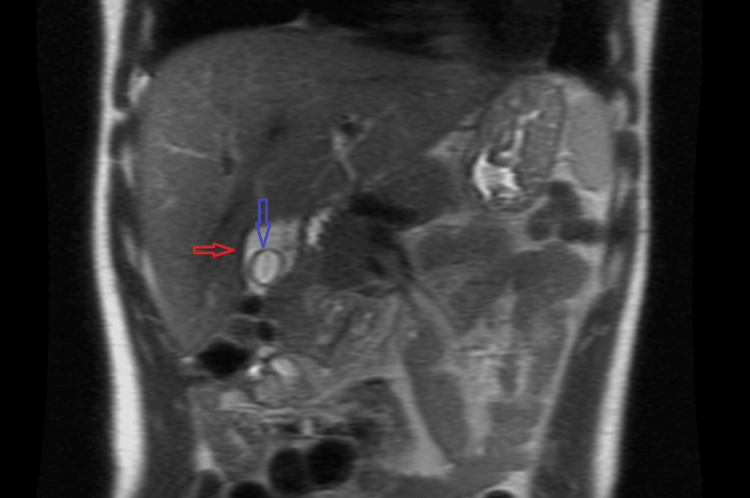
MRCP demonstrating perihepatic fluid (red) and pericholecystic fluid (blue). MRCP: Magnetic resonance cholangiopancreatography.

**Figure 2:**
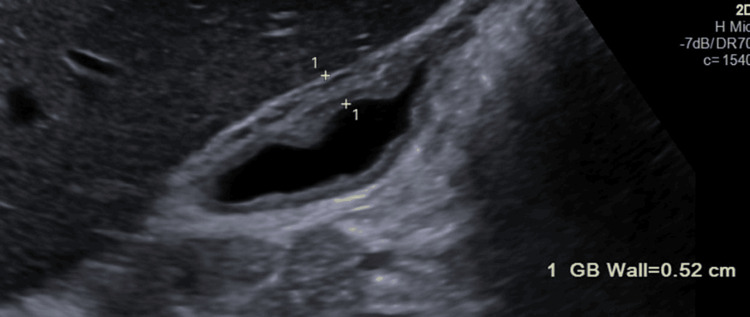
Right upper quadrant ultrasound demonstrating gallbladder wall thickening.

With regards to diagnostic testing prior to the EBV work-up, we were concerned about cholecystitis given the history of presenting illness and physical exam. Therefore, a right upper quadrant ultrasound was indicated. This ultrasound revealed gallbladder wall thickening (Figure 1) and perihepatic fluid, with a normal common bile duct measuring 4mm. Given these findings and the intermediate findings of the ultrasound, the next step was to obtain a magnetic resonance cholangiopancreatography (MRCP). The MRCP demonstrated hepatomegaly, which confirmed the gallbladder wall thickening and peri-hepatic fluid (Figure 2) but showed no evidence of biliary ductal dilation or choledocholithiasis. Blood cultures were obtained, showing no growth. Other potential infectious causes, including Lyme disease, Ehrlichia, Anaplasma, Rickettsia, and Babesia, were ruled out by PCR testing modalities.

## Discussion

The phenomenon of EBV masking acute cholecystitis is a complex clinical challenge that warrants more in-depth analysis to explore underlying pathophysiological mechanisms. To this date, there are only four case reports elucidating this phenomenon. This discussion delves into why and how EBV can obscure the clinical presentation of organic acute cholecystitis, along with the potential pathophysiological processes involved. Both EBV and acute cholecystitis consist of an overlapping clinical presentation of these two conditions. Both processes share symptoms such as fever, right upper quadrant pain, and hepatomegaly. As such, this can lead clinicians to initially consider EBV as the primary diagnosis, delaying the recognition of an underlying acute cholecystitis [1]. EBV has a predilection for the infection of hepatocytes, which induces hepatitis-like symptoms and elevated liver enzymes [2]. The hepatic involvement can, therefore, mimic primary hepatobiliary disorders, diverting clinical attention away from the gallbladder. Consequently, the typical signs and laboratory abnormalities associated with acute cholecystitis, such as gallbladder wall thickening and elevated bilirubin levels, may be erroneously attributed to EBV-induced hepatic dysfunction [3]. Innate to EBV is the ability to exert immunomodulatory effects on the host immune system, which can further blur the clinical picture. It has been suggested in the literature that EBV infections can suppress the immune response, potentially dampening inflammatory markers that are typically associated with acute cholecystitis [4]. This immune suppression may result in atypical laboratory findings such as normal to mildly elevated WBC count that could otherwise be mistaken for hemodilution. The diagnostic challenges posted by EBV infections masking acute cholecystitis highlight the need for a multidisciplinary approach that combines clinical acumen with judicious use of imaging studies. Ultrasonography remains a valuable tool in the diagnosis of cholecystitis. It may yield unequivocal results when gallbladder inflammation is overshadowed by EBV-related hepatic changes [5]. In such cases, additional imaging modalities such as MRCP or hepatobiliary scintigraphy may be necessary to confirm the diagnosis.

## Conclusions

In conclusion, EBV's ability to mask acute cholecystitis through elevations of liver enzyme tests, abnormal diagnostic tests showing perihepatic fluid, pericholecystic fluid, and gallbladder wall thickening is a complex interplay of overlapping clinical features, hepatobiliary involvement, immunomodulation, and diagnostic challenges. Healthcare providers should maintain a high index of suspicion for cholecystitis in patients with compatible symptoms, mainly when dealing with individuals positive for EBV. A comprehensive evaluation, including a detailed clinical history, physical examination, and judicious use of advanced imaging techniques, is crucial for achieving an accurate diagnosis and providing timely and appropriate management for affected patients.
